# Toxicities of PARP inhibitors in genitourinary cancers

**DOI:** 10.1097/MOU.0000000000001297

**Published:** 2025-05-07

**Authors:** János Szalontai, Tibor Szarvas, Marcin Miszczyk, Péter Nyirády, Shahrokh F. Shariat, Tamás Fazekas

**Affiliations:** a Department of Urology, Semmelweis University, Budapest, Hungary; b Department of Urology, University of Duisburg-Essen and German Cancer Consortium (DKTK)-University Hospital Essen, Essen, Germany; c Department of Urology, Comprehensive Cancer Center, Medical University of Vienna, Vienna, Austria; d Collegium Medicum – Faculty of Medicine, WSB University, Dąbrowa Górnicza, Poland; e Department of Urology, University of Texas Southwestern Medical Center, Dallas, Texas, USA; f Department of Urology, Second Faculty of Medicine, Charles University, Prague, Czech Republic; g Department of Urology, Weill Cornell Medical College, New York, New York, USA; h Karl Landsteiner Institute of Urology and Andrology, Vienna, Austria; i Research Centre for Evidence Medicine, Urology Department, Tabriz University of Medical Sciences, Tabriz, Iran; j Hourani Center for Applied Scientific Research, Al-Ahliyya Amman University, Amman, Jordan; k Centre for Translational Medicine, Semmelweis University, Budapest, Hungary

**Keywords:** adverse event, anemia, BRCA, fatigue, genetic test, niraparib, olaparib, poly ADP-ribose polymerase inhibitors, prostate cancer, side effects, talazoparib, toxicity

## Abstract

**Purpose of review:**

Recent advancements in the understanding of the genetic background of genitourinary cancers allowed for a successful introduction of targeted antitumor agents to prostate cancer (PCa) treatment. Inhibitors of the poly ADP-ribose polymerase enzyme (PARPi) transformed the treatment landscape of metastatic prostate cancer, and being increasingly studied in earlier disease stages. However, they are associated with nonnegligible toxicity, therefore, we aimed to summarize their side-effect profile in patients with PCa.

**Recent findings:**

Hematologic toxicities, particularly anemia, thrombocytopenia, and neutropenia are among the most common and serious adverse events associated with PARPi, highlighting the need for regular blood count monitoring. Nonhematologic side effects, including fatigue, nausea, vomiting, diarrhea, and constipation, are common, and can be mitigated with supportive interventions like dietary modifications, antiemetics, or stool management techniques. Special attention should be given to patients with therapy-resistant or persistent cytopenia, in whom bone marrow biopsy should be considered, as it can indicate myelodysplastic syndrome and acute myeloid leukemia.

**Summary:**

PARP inhibitors represent a major advancement in the management of metastatic prostate cancer, offering a significant survival benefit in applicable cases. However, patients need to be carefully selected and informed, to allow for optimal balancing between the benefits and nonneglectable risks of severe toxicities. Better understanding of PARPi toxicity profile can improve personalized decision-making and enhance treatment compliance, through raising patients’ awareness about the possible side effects of PARPi.

## INTRODUCTION – THE UTILITY OF POLY ADP-RIBOSE POLYMERASE INHIBITORS IN UROLOGICAL MALIGNANCIES

Owing to the recommendations against systematic screening, the incidence of metastatic prostate cancer has been steadily increasing, placing a significant burden on healthcare systems [1,2]. While androgen deprivation therapy (ADT) remains the backbone of treatment for metastatic disease, several other agents and their combinations became standard of care, including androgen receptor pathway inhibitors (ARPI), taxane chemotherapy (docetaxel, cabazitaxel), radioligands, and poly ADP-ribose polymerase inhibitors (PARPi) such as rucaparib, olaparib, and talazoparib [3]. Consequently, with the expanding treatment landscape, the opportunity for personalized therapies has improved, including the use of PARPi [4^▪▪^]. The inhibition of base-excision repair enzymes PARP 1 and 2, induces tumor cell death, particularly in cells harboring deficiencies in DNA-repair pathways, most notably the homologous recombination repair (HRR) pathway [5]. This effect, known as “synthetic lethality”, underlies the antitumor activity of PARPi. The prevalence of HRR mutations in metastatic castration-resistant prostate cancer (mCRPC) is approximately 10–28% with nearly half of these mutations involving the breast cancer gene 1 and 2 (*BRCA*); therefore PARPi represent a viable and effective treatment in advanced disease [6,7]. Another proposed mechanism of HRR deficiency is through the use of ARPI, which are hypothesized to induce a “BRCAness” state, leading to synergism between ARPI and PARPi [8^▪^,^9^^▪▪^]. Currently in the mCRPC stage, olaparib and rucaparib are approved as monotherapies, while combination therapies include olaparib with abiraterone, niraparib with abiraterone, and talazoparib with enzalutamide [3,10^▪▪^]. Notably PARPi are increasingly studied in earlier disease stages [3,11^▪^]. Considering the unique mechanism of action of PARPi, alone or in combination with ARPI, there is a compelling need to assess the toxicities associated with these agents in PCa patients, which was the aim of this review. 

**Box 1:**
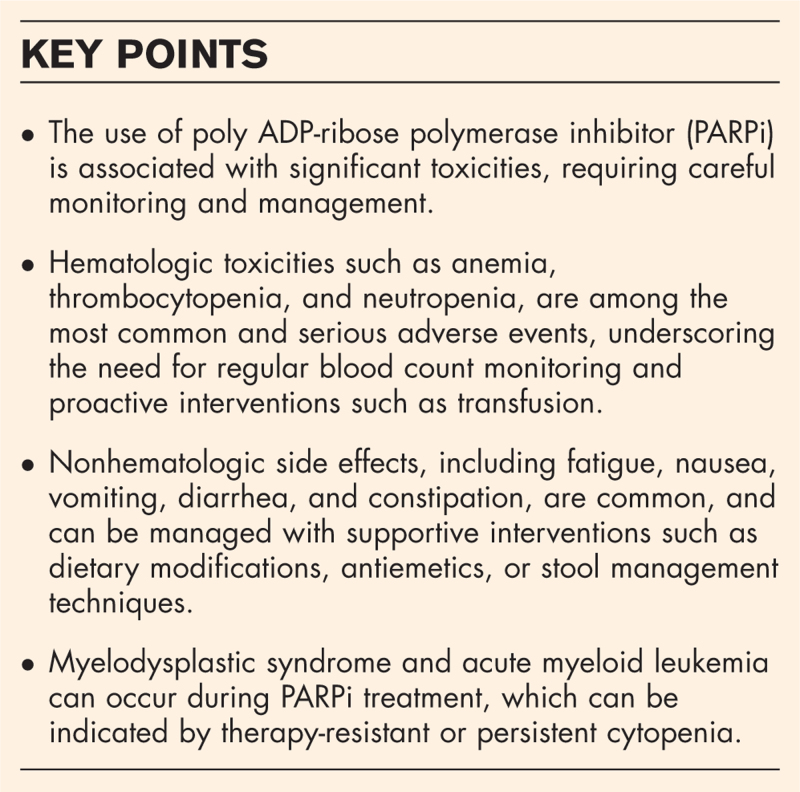
no caption available

### Tolerability of poly ADP-ribose polymerase inhibitor

PARPi monotherapy or in combination with ARPI have been shown to prolong overall and progression-free survival of metastatic castration-resistant prostate cancer patients with- or without mutations in the HRR pathway [10^▪▪^]. Two phase III randomized controlled trials (RCT), the PROfound [12^▪^,13^▪^] and the TRITON3 [14^▪^] assessed olaparib and rucaparib, respectively, in molecularly selected patients with mCRPC (Table 1). The combination of PARPi and ARPI were assessed in three phase III RCTs: PROpel (olaparib + abiraterone) [8^▪^,15^▪^], MAGNITUDE (niraparib + abiraterone) [16^▪^,17^▪^], and TALAPRO-2 (talazoparib+enzalutamide) [18^▪^,19^▪^] (Table 1). The incidence of dose reduction with PARPi ranged from 20% (PROpel) to 52% (TALAPRO-2), with higher incidence on the intervention arms in all RCTs (Table 1). Treatment discontinuation rates ranged from 14% (PROpel) to 20% (TALAPRO-2), and in most trials were higher with PARPi. However, in the TRITON3, the treatment discontinuation rate was the highest in patients receiving docetaxel in the control arm (32%) [14^▪^]. These data suggest that PARPi are tolerated less well than ARPI; however, they may be similar to or more tolerable than taxane chemotherapy.

**Table 1:** Dose reduction and treatment discontinuation rates in phase III trials assessing the efficacy of PARPi monotherapy or in combination with androgen receptor pathway inhibitors

Study	Intervention and comparator	Dose reduction	Treatment discontinuation
PROfound [12^▪^,13^▪^]	Olaparib vs. ARPI switch (abiraterone or enzalutamide)	22% vs. 18%	18% vs. 8%
TRITON3 [14^▪^]	Rucaparib vs. abiraterone/enzalutamide/docetaxel	39% vs. 25%^a^	15% vs. 22%^a^
PROpel [8^▪^,15^▪^]	Olaparib+abiraterone vs. placebo+abiraterone	20% vs. 6%	14% vs. 8%
TALAPRO-2 [18^▪^,19^▪^]	Talazoparib+enzalutamide vs. placebo+enzalutamide	52% vs. 6%	20% vs. 14%
MAGNITUDE [16^▪^,17^▪^]	Niraparib+abiraterone vs. placebo+abiraterone	20% vs. 4%	15% vs. 6%

a Overall incidence of abiraterone/enzalutamide/docetaxel control arms of the TRITON3 study.

### Adverse events of poly ADP-ribose polymerase inhibitor

In all trials, the incidence and the severity of adverse events followed a similar pattern (Table 2). While any grade adverse events were comparable between the intervention and control groups (range: 95–100% vs. 88–99%), PARPi were associated with higher rate of grade ≥3 toxicities (range: 47–75% vs. 38–53%). The most commonly reported adverse events included anemia, fatigue, nausea, vomiting, diarrhea, headache, and constipation (Table 2).

**Table 2:** Incidence of the most frequently reported adverse events in phase III trials with PARP inhibitors

Adverse event	Grade	PROfound	TRITON3	PROpel	TALAPRO-2	MAGNITUDE
Any	Any	95% vs. 88%	100% vs. 99%	97% vs. 95%	98% vs. 95%	99% vs. 94%
	≥3	51% vs. 38%	60% vs. 53%	47% vs. 38%	75% vs. 45%	56% vs. 43%
Anemia	Any	46% vs. 15%	47% vs. 18%	46% vs. 16%	65% vs. 16%	50% vs. 23%
	≥3	21% vs. 5%	24% vs. 1%	15% vs. 3%	41% vs. 5%	29% vs. 9%
Thrombocytopenia	Any	NA	19% vs. 0%	NA	25% vs. 3%	23% vs. 10%
	≥3	3% vs. 0%	6% vs. 0%	NA	7% vs. 1%	4% vs. 2%
Neutropenia	Any	NA	14% vs. 8%	NA	32% vs. 7%	15% vs. 7%
	≥3	4% vs. 0%	7% vs. 8%	NA	19% vs. 1%	5% vs. 2%
Nausea	Any	41% vs. 19%	50% vs. 19%	28% vs. 13%	21% vs. 17%	25% vs. 15%
	≥3	1% vs. 0%	3% vs. 1%	1% vs. 1%	2% vs. 1%	1% vs. 1%
Vomiting	Any	18% vs. 12%	24% vs. 8%	13% vs. 9%	NA	15% vs. 8%
	≥3	2% vs. 1%	1% vs. 1%	1% vs. 1%	NA	1% vs. 1%
Fatigue	Any	41% vs. 32%	61% vs. 63%	37% vs. 28%	33% vs. 27%	30% vs. 19%
	≥3	3% vs. 5%	7% vs. 9%	2% vs. 2%	2% vs. 1%	4% vs. vs. 5%
Diarrhea	Any	21% vs. 7%	31% vs. 28%	17% vs. 9%	12% vs. 11%	NA
	≥3	1% vs. 0%	1% vs. 2%	1% vs. 1%	0% vs. 0%	NA
Constipation	Any	18% vs. 15%	27% vs. 15%	17% vs. 14%	13% vs. 17%	33% vs. 16%
	≥3	0% vs. 0%	1% vs. 1%	0% vs. 1%	0% vs. 0%	1% vs. 0%

#### Haematological toxicity

Anemia is the most common side effect of PARPi-based treatments, with an incidence of 46–65% for any grade events and 15–41% for grade ≥3 adverse events based on data from phase III RCTs. In a real-world basket cohort of patients treated with PARPi for any cancers, the time to first onset of anemia was approximately 4 weeks. The rate of patients with indications for blood transfusion can be as high as 27% (MAGNITUDE study) [16^▪^,20^▪^]. A meta-analysis of 8 RCTs found that PARPi are associated with higher rate of any grade [risk ratio (RR): 3.37, 95% confidence interval (CI): 2.37–4.79], and severe anemia (RR: 6.94, 95% CI: 4.06–11.86) as compared to controls, in majority treated with ARPI [21]. Thrombocytopenia (any: 19–25%, grade ≥3: 3–7%) and neutropenia (any: 14–32%, grade ≥3: 4–19%) are reported less frequently (Table 2); however, the incidence of these AEs with PARPi exceeds that observed in the control arms [21]. Interestingly, among the studied PARPis, niraparib has been shown to be associated with the lowest incidence of anemia, thrombocytopenia, and neutropenia; however, no difference has been shown between PARPi monotherapy vs. combination with ARPI, and with regard to treatment duration [21]. Considering the high rate of hematologic toxicities, baseline and monthly examinations of full blood count are recommended during the first year of treatment, and at regular intervals thereafter. They allow to detect clinically significant changes in blood parameters potentially requiring transfusion, PARPi dose modification, or sometimes even treatment interruption and discontinuation [21].

#### Nausea and vomiting

Nausea and vomiting are frequently reported adverse events of PARPis, affecting 21–50% and 13–24% of patients in phase III RCTs, respectively (Table 2). They usually occur very early; the first onset in most patients is in the first two months of the treatment. Both nausea and vomiting are generally intermittent and can be successfully treated with antiemetic drugs (metoclopramide or ondansetron), dose reduction or in severe cases treatment interruption. Antiemetic prophylaxis is generally not recommended.

#### Fatigue

Fatigue is a common and bothersome symptom of patients treated with PARPis, but also a known adverse event of ADT and ARPIs, which are often administered concomitantly. In phase III RCTs, 30–61% of prostate cancer patients experienced some degree of fatigue (Table 2). In low grade fatigue, nonpharmacologic treatments such as physical activity, optimizing nutrition and diet, conservating energy (e.g.: planning activities and prioritizing), psychosocial support can be helpful. Psychostimulants can be discussed in severe cases; however, only under the rigorous supervision of the treating physician [22].

#### Diarrhea/constipation

PARPi-associated diarrhea and constipation have overall similar incidence affecting approximately 12–33% of patients each (Table 2). To manage diarrhea, dietary adjustments (such as avoiding high-fiber and spicy foods and staying hydrated with electrolyte-rich fluids) should be advised. In severe cases, antidiarrheal medications such as loperamide can be administered. For constipation, increasing dietary fiber intake, hydration, and using stool softeners or laxatives like polyethylene glycol can help to relieve the symptoms [23].

#### Myelodysplastic syndrome and acute myeloid leukemia

Myelodysplastic syndrome and acute myeloid leukemia are rare, but serious side effects of PARPis [24]. The overall incidence of these hematologic adverse events are estimated to be 0.73% [24], with a 2.63 (95% CI 1.13–6.14, *P* = 0.026) Peto odds ratio compared to placebo pooled from clinical trials of patients with a broad range of tumors [24]. The latency period from first PARPi exposure to myelodysplastic syndrome and acute myeloid leukemia was reported to be about 18 and 21 months, respectively [24]. In patients with prostate cancer treated with PARPi, recurrent, persistent, or unexplained cytopenia during follow-up should prompt suspicion of bone marrow failure syndromes and may indicate bone marrow biopsies [24].

## DISCUSSION

The increasing utility and availability of PARPi monotherapy and their combination with ARPI changed the treatment landscape of metastatic castration-resistant prostate cancer, as they prolong overall and progression-free survival, particularly for patients with BRCA and HRR mutations (Table 3) [10^▪▪^]. However, their use is associated with significant toxicities, requiring careful monitoring and management. Consequently, as the absolute treatment benefit gained with PARPis strongly depends on the underlying genetic alteration (Table 3), careful evaluation of associated risks and benefits is needed upon patient counseling. Hematologic toxicities, particularly anemia, thrombocytopenia, and neutropenia are among the most common and serious adverse events, highlighting the need for regular blood count monitoring and proactive interventions. Nonhematologic side effects, including fatigue, nausea, vomiting, diarrhea, and constipation, are common, and in the majority can be mitigated with supportive interventions like dietary modifications, antiemetics, or stool management techniques. In case of severe, therapy-resistant side effects, dose reduction or treatment interruption should be considered. Special attention should be given to patients with therapy-resistant or persistent cytopenia, as it can indicate myelodysplastic syndrome and acute myeloid leukemia, warranting bone marrow biopsy. In general, the toxicity profile and tolerability of PARPi is comparable to that of taxane chemotherapy, however, it is less favorable as compared to ARPIs. As PARPis are available from 2020 for patients with prostate cancer, insurance claim-based real-world studies are warranted in the future to assess their toxicity outside of a trial setting. Moreover, as their indication expands in earlier disease stages with longer time on treatment, focus on toxicity management will be critical to maximize the clinical benefit.

**Table 3:** Main oncologic efficacy findings from phase 3 randomized controlled trials on PARP inhibitors

Trial		Progression-free survival (95% CI)	Overall survival (95% CI)
PROfound	Cohort A (*BRCA1, BRCA2, ATM*)	HR 0.34 (0.25–0.47) 7.4 vs. 3.6 months	HR 0.42 (0.19–0.91)^a^ 19.1 vs. 14.7 months
	Cohort B (12 prespecified HRR genes)^b^	HR 0.88 (0.58–1.36) 4.8 vs. 3.3 months	HR 0.83 (0.11–5.98)^a^ 14.1 vs. 11.5 months
	Cohort A+B	HR 0.49 (0.38–0.63) 5.8 vs. 3.5	HR 0.55 (0.29–1.06)^a^ 17.3 vs. 14.0 months
TRITON3	*BRCA1, BRCA2*	HR 0.5 (0.36–0.69) 11.2 vs. 6.4 months	HR 0.81 (0.58–1.12)^c^ 24.3 vs. 20.8 months
PROpel	Regardless of genetic alteration	HR 0.66 (0.54–0.81) 24.8 vs. 16.6 months	HR 0.81 (0.67–1.00) 42.1 vs. 34.7 months
TALAPRO-2	Regardless of genetic alteration	HR 0.45 (0.33–0.61) NR vs. 13.8 months	HR 0.69 (0.46–1.03)^c^ NR vs. 33.7 months
MAGNITUDE	Patients with HRR alterations	HR 0.73 (0.56–0.96) 16.6 vs. 13.7 months	HR 0.82 (0.60–1.10)^d^
	*BRCA1, BRCA2*	HR 0.55 (0.39–0.78) 19.5 vs. 10.9 months	HR 0.54 (0.33–0.90)^d^

a After adjusting for cross-over.

b HRR genes including: *BRIP1, BARD1, CDK12, CHEK1, CHEK2, FANCL, PALB2, PPP2R2A, RAD51B, RAD51C, RAD51D, RAD54L*.

c Data immature at the time of last publication.

d After adjustment for subsequent treatments at the time of the second interim analysis.

## CONCLUSION

Considering the significant side effect profile of PARPi, patients need to be carefully selected and informed, to allow for optimal balancing between the risks and benefits associated with treatment. This can enable personalized decision-making and enhance treatment adherence and patient outcomes with PARPis.

## Acknowledgements

*Tamás Fazekas was supported by the EUSP Scholarship of the European Association of Urology (Scholarship S-2023-0006). This work was supported by the Hungarian National Eötvös Grant of the Hungarian state.*

### Financial support and sponsorship

*None.*

### Conflicts of interest

*Shahrokh F. Shariat received the following honoraria: Astellas, AstraZeneca, BMS, Ferring, Ipsen, Janssen, MSD, Olympus, Pfizer, Roche, Takeda. Consulting or advisory role: Astellas, AstraZeneca, BMS, Ferring, Ipsen, Janssen, MSD, Olympus, Pfizer, Pierre Fabre, Roche, Takeda. Speakers’ bureau: Astellas, AstraZeneca, Bayer, BMS, Ferring, Ipsen, Janssen, MSD, Olympus, Pfizer, Richard Wolf, Roche, Takeda. Tamás Fazekas received honoraria from Astellas, and support for attending meetings from Astellas, AstraZeneca, and Janssen. The other authors declare no conflicts of interest associated with this manuscript.*

## REFERENCES AND RECOMMENDED READING

Papers of particular interest, published within the annual period of review, have been highlighted as:

▪ of special interest
▪▪ of outstanding interest
